# Complications and Treatment of Early-Onset Type 2 Diabetes

**DOI:** 10.5812/ijem-135004

**Published:** 2023-08-06

**Authors:** Fahimeh Soheilipour, Naghmeh Abbasi Kasbi, Mahshid Imankhan, Delaram Eskandari

**Affiliations:** 1 Minimally Invasive Surgery Research Center, Aliasghar Children Hospital, School of Medicine, Iran University of Medical Sciences, Tehran, Iran; 2 Multiple Sclerosis Research Center, Neuroscience Institute, Tehran University of Medical Sciences, Tehran, Iran; 3 Tehran Medical Branch, Islamic Azad university, Tehran, Iran; 4 Department of Endocrinology, Rasool Akram Medical Complex, School of Medicine, Iran University of Medical Sciences, Tehran, Iran

**Keywords:** Diabetes Mellitus Type 2, Complications, Treatment, Adolescent, Adult, Pediatric

## Abstract

**Context:**

Global reports have revealed a dramatic rise in the number of patients diagnosed with type 2 diabetes (T2DM) over the past three decades in all age groups, even in children and adolescents. The physiologic phenomenon of insulin resistance during puberty, as well as genetic and epigenetic factors, are implicated in this phenomenon. It seems that patients with early-onset T2DM experience a more aggressive clinical course; however, limited treatments available for these patients pose a challenge. This narrative review intends to scrutinize the micro- and macrovascular complications and treatments of patients with early-onset T2DM.

**Methods:**

The literature search was conducted in the PubMed database to identify all relevant original English articles published from the beginning of 2018 until January 2023.

**Results:**

Vascular complications, such as albuminuria, hypertension, cardiovascular diseases, and retinopathy, were seen to be more common in early-onset T2DM compared to type 1 diabetes. The odds ratio of vascular complications was higher in early-onset compared to late-onset T2DM. In children and adolescents with T2DM, the only approved medications included metformin, insulin, and glucagon-like peptide-1 agonists. Treatment of early-onset T2DM with metformin monotherapy cannot yield durable glycemic control, and most patients need early combination therapy.

**Conclusions:**

During the past years, the frequency of early-onset T2DM has been growing at an alarming rate. Vascular complications in these patients seem more aggressive and more challenging to control. Hence, further clinical trials should be conducted to develop novel therapeutic approaches and evaluate their long-term benefits in terms of glycemic control and preventing future complications.

## 1. Context

Early-onset type 2 diabetes (EOD) refers to diabetes that occurs in younger people below the age of 40, and its prevalence has been rising in recent years. This condition is associated with a high risk of cardiovascular complications and affects people more commonly in their working years, highlighting the negative societal implications of the disease (1, 2). Physiologic insulin resistance during puberty, a family history of diabetes, obesity, sedentary lifestyles, and socioeconomic status are implicated in the current rise in the incidence of type 2 diabetes (T2DM) (3).

In youths (10 - 19 years), T2DM is considered an aggressive disease leading to adverse complications at a faster rate than type 1 diabetes (T1DM). Proliferative retinopathy was seen in 5.6% and 9.1% of children suffering from T1DM and early-onset T2DM, respectively. Meanwhile, the benefits of new medications are still a matter of debate since there are few therapeutic options available for T2DM in children and adolescents (4).

As an emergent healthcare concern in pediatrics, T2DM causes many challenges regarding the appropriate choice of treatment strategies. Medications should be started early and administered continuously. Moreover, weight control in obese individuals and screening for T2DM in high-risk children should be recommended (5). Available treatments have markedly expanded with the advent of dipeptidyl peptidase-4 (DPP4) antagonists, glucagon-like peptide 1 (GLP-1) agonists, and sodium-glucose co-transporter-2 (SGLT2) inhibitors. These novel treatments, however, still need to be officially approved for children (6). Lifestyle improvement in combination with medications like metformin, insulin, and a GLP-1 analogue, liraglutide, is currently suggested to manage these patients (7). Understanding the complications and management strategies of childhood T2DM is crucial for finding effective therapeutic plans.

To the best of our knowledge, no review has been published in the recent five years addressing the vascular consequences and therapeutic modalities of early-onset T2DM. In the present study, we intend to review the complications and treatment approaches of this disease.

## 2. Methods

### 2.1. Study Design

This narrative review was conducted to evaluate the micro- and macrovascular complications, as well as treatment strategies for early-onset T2DM.

### 2.2. Search Strategy

The literature search was conducted in the PubMed database to find relevant articles published from January 2018 to January 2023.

Boolean operators (AND & OR) were used to conduct a comprehensive search using a combination of these keywords: “Diabetes Type II”, “Diabetes Type two”, “Diabetes Type 2”, “Diabetes Adult-Onset”, “Diabetes Adult Onset”, “Type II diabetes”, “Type two diabetes”, “Type 2 diabetes”, “Non-Insulin Dependent Diabetes”, “Young-onset”, “Young onset”, “Youth-onset”, “Youth onset”, “Young*”, “Youth*”, “Adult-onset”, “Adult onset”, “Adult*”, “Young adult*”, “Young adult-onset”, “Young adult onset”, “Adolescent*”, “Adolescence”, “Adulthood”, “Teenager*”, “Pediatric*”, “Children”, “age < 40”, “Complication*”, “Micro vascular”, “Macro vascular”, “Macro-vascular”, “Micro-vascular”, “Retinopathy”, “Nephropathy”, “Neuropathy”, “Microvascular*”, “Macrovascular*”, “Therapeutic*”, “Treatment*”, “Drug*”, “Medication*”, and “Management*”.

### 2.3. Inclusion and Exclusion Criteria

English language original studies investigating the prevalence or prognosis of vascular complications or treatment of T2DM in pediatrics, young adults, and adults (age < 40) were included. Reviews, case reports, and case series were excluded. Studies that did not report any of the desired outcome measures or those assessing autoimmune or maturity-onset diabetes of the young were excluded.

## 3. Results

**Figure 1.**
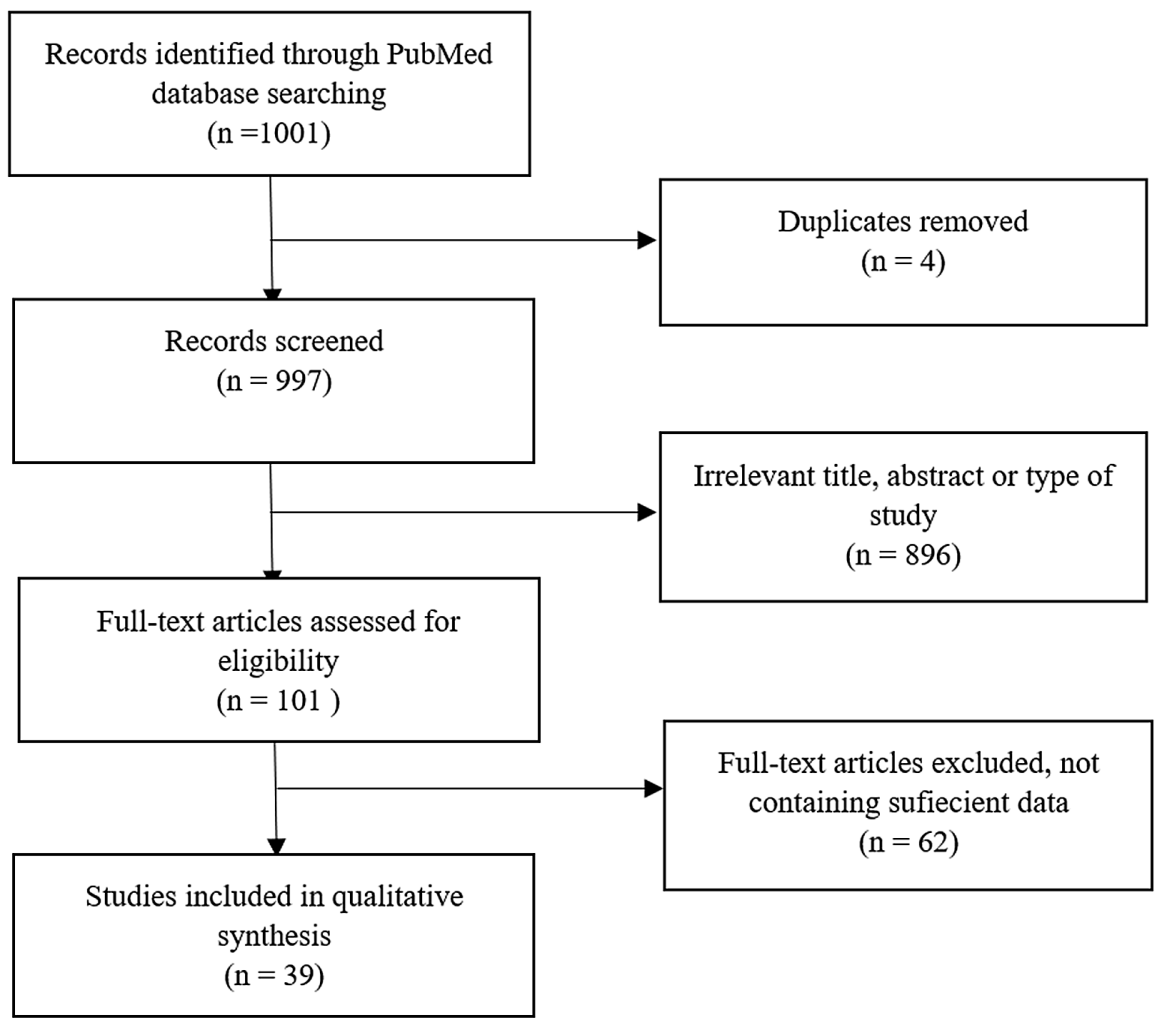
The PRISMA flow chart of the study

A total of 1001 articles were evaluated. Duplicates were excluded. A total of 101 relevant articles were chosen after reading the title/abstract. Finally, 39 articles were reviewed by reading the full text. A visual description of our literature search has been provided in Figure 1.

### 3.1. Complications

#### 3.1.1. Early-Onset T2DM

According to a study on young patients with T2DM, vascular complications were reported as 37.1% retinopathy, 34.8% nephropathy, 34.8% neuropathy, 53.9% hypertension, 78.7% dyslipidemia, and 7.9% established cardiovascular disease (8). According to another report, among 193 children with T2DM, nine patients had at least one diabetes-related complication at the time of diagnosis (9).

Microvascular complications of T2DM increased from 50% at nine years of follow-up to 80.1% after 15 years of follow-up in youths with a mean age of 26 years (10). A significant decline was seen in the diastolic function of adolescents with T2DM, who had the highest prevalence of cardiac abnormalities, such as left ventricular hypertrophy (LVH). The factors predicting an adverse prognosis included hypertension, obesity, female gender, Hispanic and non-Hispanic black ethnicity, poor glycemic maintenance, and tachycardia (11).

Considering the complications of T2DM in patients diagnosed at the age of < 45 years, those with severe insulin deficiency were more likely to develop nephropathy and diabetic retinopathy (DR); however, neuropathy was more prevalent in patients with mild insulin deficiency (12).

Young people with T2DM were shown to have an increase in inner and entire retinal thickness, which was associated with higher fasting glucose and glycosylated hemoglobin (HbA1c) levels (13). A study reported a frequency of 49% for DR in youths with T2DM. The risk factors of DR progression comprised a lower body mass index (BMI), greater HbA1c levels, hypertension, elevated triglyceride (TG) levels, and the presence of other comorbidities. However, multivariate analysis revealed the poor control of HbA1c as the main contributor to the development and progression of DR (14).

The risk of diabetic peripheral neuropathy (DPN) was more significant in males, older patients, and those with a greater BMI (15).

Albuminuria was frequent in adolescents with T2DM. Each 1% rise in HbA1c carried a greater risk of albuminuria, hyperfiltration, and a rapid decrease in estimated glomerular filtration rate (eGFR). Higher systolic blood pressure and uric acid (UA) levels and lower beta cell functional indices raised the risk of microalbuminuria. Elevated TG levels also exaggerated the risk of albuminuria (16, 17). A significant relationship was noticed between early-onset T2DM and the activity of the renin-angiotensin system, which was consistent with poor glycemic control and early-onset albuminuria (18).

#### 3.1.2. Early-Onset T2DM vs Late-Onset T2DM

Type 2 diabetes patients with EOD represent a higher risk of complications compared to late-onset diabetes (LOD) (onset of diabetes at age > 40 years). However, the probability of macrovascular complications was comparable between EOD and LOD after adjustment according to propensity score matching. The odds ratio of developing microvascular complications was reported to be higher in EOD (19).

Patients with EOD seem to have lower systolic blood pressure (SBP) and greater BMI, waist circumference, fasting plasma glucose, UA, TG, total cholesterol (TC), and HbA1c. The incidence of DR was significantly higher in the EOD group, while DPN was less frequent among EOD patients. Also, UA, length of diabetes, and SBP were independent risk factors for EOD-associated nephropathy (20-23). In young patients, the average duration to develop atherosclerotic cardiovascular disease (ASCVD) and arrhythmogenic cardiomyopathy (ACM) was 9.1 years in high-risk patients and 10.0 years in low-risk patients (23).

Patients with early-onset T2DM had significantly higher death rates and vascular complications (stroke, heart failure, myocardial infarction), as well as a higher incidence of DR and a lower risk of chronic kidney disease (CKD) (24, 25).

Diabetic retinopathy was also more frequent in EOD patients compared to those with LOD (26). A study showed a higher prevalence of DR, CKD, neuropathy, and carotid artery plaque in EOD patients. After adjustment for the duration of the disease, neuropathy remained the only complication that was more prevalent in EOD patients. Forty-seven percent of patients with EOD and 23% of those with LOD were classified into the severe insulin-deficient group representing the highest risk of retinopathies (27). Regarding the prognosis of proliferative DR between LOD and EOD patients, those in the latter group showed more adverse visual outcomes and a greater probability of post-operative vitreous hemorrhage (2).

#### 3.1.3. Early-Onset T2DM vs T1DM

Based on studies on youths with T1DM and T2DM, patients with T2DM had higher BMIs, blood pressure, HbA1c, cholesterol, median five-year cardiovascular risk, and more severe dyslipidemia. Also, inflammatory markers, albuminuria, and autonomic and peripheral nerve abnormalities were more pronounced in this group (28, 29).

Children with T2DM (age ≤ 15 years) had lower HbA1c levels at the time of diagnosis, as well as higher BMIs and a greater risk of hypertension and microalbuminuria than counterparts with T1DM (30). Early-onset T2DM was associated with a higher incidence of cardiovascular autonomic neuropathy (CAN), cardiac abnormalities, LVH, peripheral neuropathy, obesity, hypertension, and albuminuria, and also a higher median level of HbA1c and longer disease duration than those with T1DM (31-34). A study on patients with age < 25 years showed higher TG levels and SBP among patients with early-onset T2DM (34).

A study on adolescents showed that end-stage kidney disease (ESKD) was seen in 6.1% of T1DM and 4.1% of T2DM patients. Further, T1DM patients were younger when ESKD was diagnosed, and in the follow-up, a higher mortality rate was noticed in the T2DM group (35). However, in another study, ESKD was noted to develop more quickly among T2DM patients with a diagnosis age of 15 - 35 years, while the prognosis and mortality rate were identical in both groups (T1DM vs. T2DM) (36). A study on young diabetic patients demonstrated no significant difference between T1DM and T2DM patients regarding the prevalence of DR, and the length of diabetes and HbA1c levels were identified as the main risk factors for DR (37).

One study found that after a 5-year follow-up post-diagnosis, 7.7% and 24.6% of youths with T2DM developed microalbuminuria and retinopathy, respectively, while these values were 3.8% for albuminuria and 19.2% for retinopathy among adolescents with T1DM. The incidence of these complications showed an increase after ten years of follow-up, and adolescents with T2DM were at a significantly greater risk of retinopathy and microalbuminuria (38). Studies’ characteristics and results regarding the complications of early-onset T2DM have been shown in Table 1.

**Table 1.** Review Articles Related to the Treatment of Early-Onset Type 2 Diabetes Mellitus

References	Study Design	Number of Participants	Age ^a^	Outcome Measures	Results
**Ke et al. (** 2 **)**	Case-control	39 T2DM with PDR (diagnosed ≤ 40)	44.6 ± 8.51	PDR	The poor visual prognosis was greater in early-onset T2DM.
		35 T2DM with PDR (diagnosed > 40)	57.7 ± 7.53		
**Lascar et al. (** 8 **)**	Cohort	100 T2DM (diagnosed < 40)	Diagnosis: 32.5 ± 5.5	Vascular complications	Vascular complications were prevalent in early-onset T2DM.
**Curran et al. (** 9 **)**	Letter	193 T2DM (age < 16)		Clinical characteristics	Nine children had at least one diabetes complication at diagnosis.
**TODAY Study Group et al. (** 10 **)**	Clinical trial	500 T2DM (adolescents)	26.4 ± 2.8	Vascular complications	The risk of microvascular complications increased over time.
**The TODAY Follow-Up Study et al. (** 11 **)**	Cohort	411 T2DM (adolescents); 194 obese controls; 51 normal-weight controls	23	Cardiac functions	Cardiac structural abnormalities were higher in adolescents with T2DM.
**Prasad et al. (** 12 **)**	Cross-sectional	1612 T2DM (diagnosed < 45)	Diagnosis: 37	Vascular complications	Retinopathy and nephropathy were more frequent in severe insulin-deficient and neuropathy in MOD.
**Mititelu et al. (** 13 **)**	Cohort	344 T2DM (adolescents)	25.0 ± 2.4	Retinal thickness alterations	Youths with T2DM showed an increase in retinal thickness.
**TODAY Study Group (** 14 **)**	Cohort	367 T2DM (adolescents)	25.4 ± 2.5	Risk factors of DR progression	HbA1c was the main factor affecting DR progression.
**TODAY Study Group (** 15 **)**	Cohort	674 T2DM (adolescents)	14	DPN	Older age, male gender, greater BMI, and HbA1c were related to DPN risk.
**TODAY Study Group (** 16 **)**	Cohort	677 T2DM (adolescents)	14	Diabetic nephropathy	Each 1% rise in HbA1c carried a greater risk of albuminuria, hyperfiltration, and a decrease in eGFR.
**Dart et al. (** 17 **)**	Cohort	187 T2DM (age 10 - 25)	15	Renal complications	Albuminuria was frequent in young indigenous people with T2DM.
**Dart et al. (** 18 **)**	Cross-sectional	183 T2DM (age 10 - 24)	14 - 15	Renal complications	Early-onset T2DM was significantly associated with renin-angiotensin system activation, which was related to higher HbA1c levels.
		100 non-diabetic controls	14.2 ± 3.2		
**Huang et al. (** 19 **)**	Cohort	260 T2DM (diagnosed ≤ 40)	53.08 ± 6.37	Vascular complications	Younger age of T2DM onset carried a greater risk of microvascular complications.
		179 T2DM (diagnosed ≤ 60)	75.24 ± 3.88		
		520 T2DM (diagnosed > 40)	53.44 ± 6.08		
		358 T2DM (diagnosed > 40)	75.27 ± 3.81		
**Huang et al. (** 20 **)**	Cross-sectional	340 T2DM (diagnosed ≤ 40)	33.3 ± 6.3	Microvascular complications	DR was more common, and DPN was less prevalent in early-onset T2DM.
		1081 T2DM (diagnosed > 40)	53.2 ± 8.5		
**Barker et al. (** 21 **)**	Cross-sectional	196 T2DM (diagnosed < 40)	46	Risk of cardiovascular complications	Patients diagnosed at a younger age had higher BMIs, waist circumference, HbA1c, and risk of cardiovascular events.
		846 T2DM (diagnosed 40 - 59)	61		
		367 T2DM (diagnosed > 60)	71		
**Baek et al. (** 22 **)**	Cohort	296 T2DM (age < 40)	31.5 ± 6.6	Vascular complications	Patients with EOD had greater levels of blood sugar at diagnosis, poor glycemic control, and a higher risk of complications.
		2,154 T2DM (age 40 - 65)	54.4 ± 6.5		
		1,029 T2DM (age ≥ 65)	71.3 ± 5.4		
**Koye et al. (** 23 **)**	Cohort	29678 T2DM (age 18 to 39)	33 ± 5	Macrovascular complications	Early-onset T2DM carried the same cardiovascular and mortality risks as late-onset T2DM, regardless of cardiometabolic risk factors at diagnosis.
		56798 T2DM (age 40 to 49)	45 ± 3		
		93698 T2DM (age 50 to 59)	55 ± 3		
		107261 T2DM (age 60 to 69)	64 ± 3		
		83419 T2DM (age 70 to 79)	74 ± 3		
**Yen et al. (** 24 **)**	Cohort	5902 T2DM (diagnosed 18 - 40)	33.2 ± 5.1	Vascular complications	Early-onset T2DM represented a significantly higher risk of mortality and macro- and microvascular complications.
		32605 T2DM (diagnosed 40 - 60)	50.6 ± 5.37		
		28013 T2DM (diagnosed 60 - 90)	70.12 ± 7.34		
		66520 non-diabetic controls			
**Wang et al. (** 25 **)**	Cohort	60 T2DM (age ≤ 44)	38.55 ± 4.97	Diabetic nephropathy	Adolescents had higher eGFR, retinopathy, and a lower risk of chronic kidney disease.
		187 T2DM (age 45 - 59)	51.13 ± 3.85		
		68 T2DM (age ≥ 60)	64.60 ± 3.22		
**Middleton et al. (** 26 **)**	Cross-sectional	348 T2DM (diagnosed 15 - 40)	48	Vascular complications	DR was more common in early-onset T2DM.
		588 T2DM (diagnosed 40 - 50)	58.6		
		796 T2DM (diagnosed 50 - 60)	67.3		
		460 T2M (diagnosed 60 - 70)	77		
**Cho et al. (** 27 **)**	Cross-sectional	1,791 T2DM (diagnosed < 40)	45.4 ± 10.6	Vascular complications	EOD carried a higher risk of neuropathy after adjustment for diabetes duration.
		8,656 T2DM (diagnosed ≥ 40)	60.5 ± 8.8		
**Aulich et al. (** 28 **)**	Case-control	134 T1DM	15.8	Vascular complications	Albuminuria and autonomic and peripheral nerve abnormalities were greater in adolescents with T2DM.
		32 T2DM	15.1		
		32 cystic fibrosis-related diabetes	14.6		
		48 controls	14.0		
**Wijayaratna et al. (** 29 **)**	Cross-sectional	1350 T2DM (age < 40, diagnosed 15 - 30)	33	Risk factors for CVD	The median five-year CVD risk score was greater in T2DM.
		731 T1DM	30		
**Haynes et al. (** 30 **)**	Cohort	2209 T1DM (age ≤ 15)	Diagnosis: 8.5 ± 4	Clinical features	Early-onset T2DM showed a greater risk of hypertension, high cholesterol, and microalbuminuria.
		229 T2DM (age ≤ 15)	Diagnosis: 12.7 ± 2		
**Carino et al. (** 31 **)**	Cohort	322 T2DM (age 10 - 18)	14.8 ± 2.3	Clinical characteristics	The prevalence of obesity, hypertension, left ventricular hypertrophy, albuminuria, and hyperfiltration was higher in T2DM.
		199 T1DM (age 10 - 18)	14.4 ± 1.7		
**Varley et al. (** 32 **)**	Cohort	66 T2DM (age < 20)	15.4	CAN	CAN was more frequent in early-onset T2DM.
		1153 T1DM (age < 20)	16.5		
**Jaiswal et al. (** 33 **)**	Cohort	1646 T1DM (diagnosed < 20)	18 ± 4	CAN	CAN was more prevalent in early-onset T2DM. Its associated factors were elevated TG and urinary Alb.
		252 T2DM (diagnosed < 20)	22 ± 4		
**Yeow et al. (** 34 **)**	Cohort	76 T1DM (age < 25)	20.4 ± 3.9	Vascular complications	Patients with early-onset T2DM showed a higher risk of cardiovascular disease, higher BMIs, hypertension, dyslipidemia, and premature nephropathy.
		24 T2DM (age < 25)	20.7 ± 3.6		
**Pleniceanu et al. (** 35 **)**	Cohort	1183 T1DM (adolescents)	18.0 ± 1.4	ESKD	T1DM and T2DM in adolescents raised the risk of ESKD. The mortality rate of T2DM was higher.
		196 T2DM (adolescents)	18.6 ± 1.7		
		1,499,143 non-diabetics (adolescents)	17.7± 1.1		
**Middleton et al. (** 36 **)**	Cohort	1248 T1DM (diagnosed 15 - 35); 1534 T2DM (diagnosed 15 - 35)		ESKD	The need for renal replacement therapy was higher in early-onset T2DM. Both groups performed equally poorly after ESKD.
**Ferm et al. (** 37 **)**	Cross-sectional	1640 DM (age 5 - 21); 1216 T1DM (age 5 - 21); 416 T2DM (age 5 - 21)	15.7 ± 3.6	DR	The prevalence of DR was similar between the groups.
**Ek et al. (** 38 **)**	Cohort	1413 T2DM (diagnosed 10 - 25); 3748 T1D (diagnosed 10 - 25)		Vascular complications	Early-onset T2DM showed a higher risk of microalbuminuria and retinopathy.

Abbreviations: T2DM, type 2 diabetes mellitus; T1DM, type 1 diabetes mellitus; DR, diabetic retinopathy; DPN, diabetic peripheral neuropathy; LOD, late-onset diabetes; CVD, cardiovascular disease; eGFR, estimated glomerular filtration rate; BMI, body mass index; PDR, proliferative diabetic retinopathy; MOD, mild obesity-related diabetes; ESKD, end-stage kidney disease; EOD, early-onset diabetes; CAN, cardiovascular autonomic neuropathy; TG, triglyceride; HbA1c, hemoglobin.

^a^ Ages are presented as mean ± SD, mean or range.

### 3.2. Treatment

In a study on early-onset T2DM, 36% of the patients were treated with oral anti-diabetic agents (OAD), 2.2% with insulin, and 60.7% with OAD plus insulin. The mean length from disease onset to the commencement of insulin therapy was 4.5 ± 3.5 years, and insulin was prescribed in the first three years after diagnosis in 52.8% of the patients (8).

A clinical trial on patients with early-onset T2DM from 2004 to 2011 compared the benefits of metformin monotherapy, metformin plus rosiglitazone, and metformin plus lifestyle modifications. After completion of the trial, the participants were transitioned to metformin with or without insulin and were enrolled in an observational follow-up study from 2011 to 2020. At least one complication occurred in 60.1% of the participants, and at least two complications developed in 28.4% of them. Patients with two or three complications during the follow-up were mainly treated with metformin alone. No adverse events were recorded during the follow-up (10).

According to another study, surgery for obese adolescents (BMI ≥ 35 kg/m^2^) with T2DM was linked to considerable weight loss, improved glycemic control, reductions in cardiovascular risk factors, and improvements in renal function. The mean HbA1c level also reduced from 6.8% to 5.5% in the patients undergoing bariatric surgery and increased from 6.4% to 7.8% in those receiving pharmaceutical therapy (39).

Another clinical trial revealed that the rate of glycemic control failure was considerably lower in young individuals treated with metformin plus rosiglitazone than in those treated with metformin alone or metformin plus intensive lifestyle counseling. After permanently discontinuing rosiglitazone to estimate its long-term efficacy, the rate of glycemic control failure did not change considerably amongst the subjects managed with the original treatment during the follow-up (40).

A clinical trial evaluated the effects of a single oral dose of empagliflozin (an inhibitor of sodium-glucose co-transporter-2) at doses of 5, 10, and 25 mg in young adults with T2DM, leading to average reductions of 0.9, 0.9, and 1.1 mmol/L in fasting blood sugar, respectively. No major adverse reaction was seen, indicating the potential of empagliflozin to be evaluated in a phase III trial in pediatrics with T2DM (41).

In another clinical trial, patients with T2DM (the age range of 10 - 24 years) were randomly allocated to receive either 10 mg of oral dapagliflozin or a placebo for 24 weeks, followed by prescribing dapagliflozin for all patients for 28 weeks in the context of an open-labeled safety extension. The average alteration in HbA1c was -0.25% in the dapagliflozin group and 0.50% in the placebo group. Sixty-nine percent of the patients in the dapagliflozin group experienced adverse reactions during the first 24 weeks of the study. However, there was no significant adverse reaction during this period in the placebo group. Only 28% of the patients in the placebo group experienced hypoglycemia (42).

Another 54-week clinical trial on adolescents with T2DM compared the benefits of treatment with 100 mg sitagliptin per day with that of placebo/metformin, showing no significant differences in HbA1c levels after 20 and 54 weeks of treatment (43).

In another clinical trial, the effects of liraglutide and metformin were assessed in adolescents with T2DM. The mean HbA1c level in the liraglutide group reduced significantly in comparison with the placebo group. Also, fasting blood sugar was reduced in the liraglutide group but raised in the placebo group. The number of patients who experienced adverse reactions in both groups was comparable; however, a greater rate of adverse events was recorded among the patients treated with liraglutide (44).

In another study, adolescents with T2DM were randomly prescribed once-weekly 2 mg exenatide or a placebo. After 24 weeks, the minimum alteration in HbA1c was -0.36% in the exenatide group and +0.49% in the placebo group. Differences from the baseline in fasting glucose, systolic blood pressure, and body weight were not significant. Sixty-one percent of the patients in the exenatide group experienced adverse reactions. Overall, the injection of exenatide once per week was shown to reduce HbA1c levels in adolescents who were sub-optimal responders to other treatments (45). The details and results of the studies investigating the treatments of early-onset T2DM are shown in Table 2.

**Table 2.** Characteristics of the Selected Articles and Their Reported Results Regarding the Complications of Early-Onset T2DM

Studies	Study Design	Number of Participants	Age ^a^	Outcome Measures	Results
**Lascar et al. (** 8 **)**	Cohort	100 T2DM (diagnosed < 40)	Diagnosis: 32.5 ± 5.5	Treatment of T2DM (young adults)	The mean duration from disease onset to the start of insulin therapy was 4.5 ± 3.5 years.
**TODAY Study Group et al. (** 10 **)**	Clinical trial	500 T2DM (young-onset)	26.4 ± 2.8	Treatment of early-onset T2DM	Treatment with metformin alone was associated with a higher rate of complications.
**Inge et al. (** 39 **)**	Cohort	30 obese T2DM (age < 19)	16.9 ± 1.3	Medical and surgical treatment	Better glycemic control was seen in severely obese adolescents with T2DM managed by surgery.
		63 obese T2DM (age 10 - 17)	15.3 ± 1.3		
**TODAY Study Group (** 40 **)**	Cohort	572 T2DM (youth-onset)	-	Glycemic failure rate	Glycemic failure rates in metformin, metformin + lifestyle modification, and metformin + rosiglitazone groups were 65.5 %, 59.4%, and 56.8%, respectively.
**Laffel et al. (** 41 **)**	Clinical trial	27 T2DM (age 10 - 17)	14.1 ± 2.0	Empagliflozin efficacy as a treatment for T2DM (young adults)	Exposure-response correlations were the same as adults. There was no major side effect.
**Shankar et al. (** 43 **)**	Clinical trial	190 T2DM (age 10 - 17)	14.0 ± 2.0	Sitagliptin therapy in T2DM patients (adolescents)	No improvement in glycemic control was seen in the sitagliptin compared to the placebo group.
**Tamborlane et al. (** 44 **)**	Clinical trial	134 T2DM (age 10 - 17)	14.6	Liraglutide plus metformin (adolescents with T2DM)	Liraglutide improved glycemic control in adolescents with T2DM.
**Tamborlane et al. (** 42 **)**	Clinical trial	72 T2DM (age 18 - 24)	16.1 ± 3.3	Dapagliflozin efficacy in T2DM patients (pediatrics, youths, and adults)	An improvement in HbA1c level was observed. The safety profile was excellent, with no major adverse reactions.
**Tamborlane et al. (** 45 **)**	Clinical trial	72 T2DM (age 10 - 18)		Exenatide injections in T2DM patients (adolescents)	Exenatide injection for 24 weeks (once per week) reduced HbA1c levels in adolescents with T2DM.

Abbreviations: T2DM, type 2 diabetes mellitus; T1DM, type 1 diabetes mellitus.

^a^ Ages are presented as mean ± SD or mean.

## 4. Discussion

According to the studies focusing on T1DM and T2DM (early-onset and late-onset), patients with early-onset disease have shown an increase in the 5-year risk of CVDs (29) and a higher incidence of vascular complications (28-34, 38).

Higher BMIs, SBP, and TG levels have been reported in patients with early-onset T2DM compared to patients suffering from T1DM (28, 30-32). In a study, patients with T1DM were at a higher risk of developing ESKD at a younger age; however, the mortality rate of T2DM patients was higher during the follow-up (35).

Regarding the socioeconomic aspect, a higher ratio of T2DM patients lived in deprived areas, which was associated with more aggressive disease progression (29, 31).

Based on articles comparing late- and early-onset T2DM, patients with early-onset disease had lower SBP, a longer period of diabetes, greater levels of serum UA, fasting plasma glucose, HbA1c, TC, and TG, as well as higher BMIs. The proportions of patients with DR, CKD, carotid artery plaque, and neuropathy were also more prominent among patients with early-onset T2DM (21, 22, 25-27).

In addition to the higher incidence of retinopathy in EOD, the visual prognosis seems to be poor in these patients, with a greater rate of recurrent postoperative vitreous hemorrhage (2). The odds ratios of DN, CKD, and DPN were greater in this group (19). In addition to microvascular complications, heart failure, stroke, and myocardial infarction seemed to be higher in those with early-onset T2DM (24).

Microvascular complications in patients with EOD were reported in 50% of the patients after nine years of follow-up and 80% after 15 years (10). The frequency of DR among youths with T2DM was reported at 49%, and its progression was associated with higher HbA1c levels. Moreover, it was reported that retinal thickening was more frequent in young people with T2DM, correlating with fasting glucose and HbA1c levels (13, 15).

Another analysis found that every 1% rise in HbA1c was correlated with a greater risk of albuminuria, hyperfiltration, and a quick fall in eGFR (16). There was also an association between early-onset T2DM and the activation of the renin-angiotensin system, which predicted poor glycemic control and early-onset albuminuria (18).

Diastolic function and myocardial remodeling were reported to deteriorate in young T2DM patients from adolescence to early adulthood (11). Choosing an appropriate treatment for T2DM patients of younger ages poses a challenge among physicians. Lifestyle modification, along with metformin administration, was suggested as the first-line treatment for adolescents with T2DM and non-prominent hyperglycemia (46).

In obese adolescents with T2DM, surgical treatment can be a reasonable choice since a significant reduction in weight can lead to better glycemic control, mitigating the risk of cardiovascular and renal diseases in the future (10, 39). It was reported that more than half of early-onset T2DM patients began insulin therapy within the first three years after diagnosis. Research on adults with T2DM shows that starting insulin therapy in the early stages of the disease can decrease the probability of developing vascular complications (8, 19).

Injecting exenatide, a GLP-1 agonist, after 24 weeks (once per week) reduced HbA1c levels in adolescents with T2DM who were sub-optimally managed with other treatments (45). Liraglutide, another GLP-1 agonist, has been recently approved for patients the age of ten years and higher. This drug has been reported to significantly reduce HbA1c and should be considered second-line therapy. The main advantage of liraglutide, respective to metformin for treating T2DM in adolescents, includes better glycemic control (4, 44, 46).

A single oral dose of empagliflozin, an SGLT-2 inhibitor, showed the same exposure response in young adults and adults with T2DM (41). Moreover, the benefits of dapagliflozin in treating T2DM in pediatrics, youths, and adults include a significant reduction in HbA1c and an excellent safety profile (42). Nevertheless, the administration of 100 mg sitagliptin (a DPP-4 inhibitor) per day exhibited no improvement in glycemic control in adolescents (43).

According to TODAY Study, young patients with T2DM presenting with two or three complications during the follow-up period were mainly treated with metformin, while patients with no complications were mostly treated with metformin plus rosiglitazone (thiazolidinediones). However, the effects of adding rosiglitazone on glycemic control were transient and disappeared after the drug was discontinued in adolescents. Since the only approved medications for early-onset T2DM are metformin, insulin, and GLP-1 agonists, the management of this condition is more complex in this population (10, 16, 40).

### 4.1. Areas of Uncertainty

Further studies and trials should be conducted to evaluate the natural course of T2DM, especially in children and adolescents. Furthermore, the long-term benefits of different therapeutic regimens, such as SGLT2 inhibitors, DPP-4 inhibitors, and other novel therapies, should be evaluated by conducting more clinical trials.

## 5. Conclusions

In the last five years, the number of patients diagnosed with early-onset T2DM has increased considerably. These patients seem to experience more severe and aggressive vascular complications, which are more challenging to control. Currently, medications other than metformin, insulin, extended-release exenatide, and liraglutide are not suggested to be used for treating early-onset T2DM in adolescents except in clinical trials. It is crucial to conduct further clinical trials to develop novel therapeutic approaches.

ijem-21-3-135004-s001.pdf

## References

[1] Lascar N, Brown JEP, Pattison H, Barnett AH, Bailey CJ, Bellary S (2018). Type 2 diabetes in adolescents and young adults.. Lancet Diabetes Endocrinol..

[2] Ke D, Hong Y, Jiang X, Sun X (2022). Clinical Features and Vitreous Biomarkers of Early-Onset Type 2 Diabetes Mellitus Complicated with Proliferative Diabetic Retinopathy.. Diabetes Metab Syndr Obes..

[3] Akinci G, Savelieff MG, Gallagher G, Callaghan BC, Feldman EL (2021). Diabetic neuropathy in children and youth: New and emerging risk factors.. Pediatr Diabetes..

[4] Rao G, Jensen ET (2020). Type 2 Diabetes in Youth.. Glob Pediatr Health..

[5] Grondahl MFG, Johannesen J, Kristensen K, Knop FK (2021). Treatment of type 2 diabetes in children: what are the specific considerations?. Expert Opin Pharmacother..

[6] Yau M, Sperling MA, Feingold KR, Anawalt B, Blackman MR, Boyce A, Chrousos G, Corpas E (2000). Treatment of Diabetes Mellitus in Children and Adolescents.. Endotext [Internet]..

[7] Serbis A, Giapros V, Kotanidou EP, Galli-Tsinopoulou A, Siomou E (2021). Diagnosis, treatment and prevention of type 2 diabetes mellitus in children and adolescents.. World J Diabetes..

[8] Lascar N, Altaf QA, Raymond NT, E. P. Brown J, Pattison H, Barnett A (2019). Phenotypic characteristics and risk factors in a multi-ethnic cohort of young adults with type 2 diabetes.. Curr Med Res Opin..

[9] Curran JA, Haynes A, Davis EA (2020). Clinical characteristics of Western Australian children diagnosed with type 2 diabetes before 10 years of age.. Med J Aust..

[10] Bjornstad P, Drews KL, Caprio S, Gubitosi-Klug R, Nathan DM, TODAY Study Group (2021). Long-Term Complications in Youth-Onset Type 2 Diabetes.. N Engl J Med..

[11] Gidding SS, Braffett BH, Shah RD, Lima J, The TODAY Follow-Up Study, TODAY Study Group (2020). Longitudinal Changes in Cardiac Structure and Function From Adolescence to Young Adulthood in Participants With Type 2 Diabetes Mellitus: The TODAY Follow-Up Study.. Circ Heart Fail..

[12] Prasad RB, Asplund O, Shukla SR, Wagh R, Kunte P, Bhat D (2022). Subgroups of patients with young-onset type 2 diabetes in India reveal insulin deficiency as a major driver.. Diabetologia..

[13] Mititelu M, Uschner D, Doherty L, Bjornstad P, Domalpally A, Drews KL (2022). Retinal Thickness and Morphology Changes on OCT in Youth with Type 2 Diabetes: Findings from the TODAY Study.. Ophthalmol Sci..

[14] TODAY Study Group (2021). Development and Progression of Diabetic Retinopathy in Adolescents and Young Adults With Type 2 Diabetes: Results From the TODAY Study.. Diabetes Care..

[15] TODAY Study Group (2021). Risk Factors for Diabetic Peripheral Neuropathy in Adolescents and Young Adults With Type 2 Diabetes: Results From the TODAY Study.. Diabetes Care..

[16] TODAY Study Group (2021). Effects of Metabolic Factors, Race-Ethnicity, and Sex on the Development of Nephropathy in Adolescents and Young Adults With Type 2 Diabetes: Results From the TODAY Study.. Diabetes Care..

[17] Dart AB, Wicklow B, Blydt-Hansen TD, Sellers EAC, Malik S, Chateau D (2019). A Holistic Approach to Risk for Early Kidney Injury in Indigenous Youth With Type 2 Diabetes: A Proof of Concept Paper From the iCARE Cohort.. Can J Kidney Health Dis..

[18] Dart AB, Wicklow B, Scholey J, Sellers EA, Dyck J, Mahmud F (2020). An evaluation of renin-angiotensin system markers in youth with type 2 diabetes and associations with renal outcomes.. Pediatr Diabetes..

[19] Huang L, Wu P, Zhang Y, Lin Y, Shen X, Zhao F (2022). Relationship between onset age of type 2 diabetes mellitus and vascular complications based on propensity score matching analysis.. J Diabetes Investig..

[20] Huang JX, Liao YF, Li YM (2019). Clinical Features and Microvascular Complications Risk Factors of Early-onset Type 2 Diabetes Mellitus.. Curr Med Sci..

[21] Barker MM, Zaccardi F, Brady EM, Gulsin GS, Hall AP, Henson J (2022). Age at diagnosis of type 2 diabetes and cardiovascular risk factor profile: A pooled analysis.. World J Diabetes..

[22] Baek HS, Park JY, Yu J, Lee J, Yang Y, Ha J (2022). Characteristics of Glycemic Control and Long-Term Complications in Patients with Young-Onset Type 2 Diabetes.. Endocrinol Metab (Seoul)..

[23] Koye DN, Ling J, Dibato J, Khunti K, Montvida O, Paul SK (2020). Temporal Trend in Young-Onset Type 2 Diabetes-Macrovascular and Mortality Risk: Study of U.K. Primary Care Electronic Medical Records.. Diabetes Care..

[24] Yen FS, Lo YR, Hwu CM, Hsu CC (2021). Early-onset type 2 diabetes <60 years and risk of vascular complications.. Diabetes Res Clin Pract..

[25] Wang J, Zhao L, Zhang J, Wang Y, Wu Y, Han Q (2020). Clinicopathologic Features and Prognosis of Type 2 Diabetes Mellitus and Diabetic Nephropathy in Different Age Groups: More Attention to Younger Patients.. Endocr Pract..

[26] Middleton TL, Constantino MI, Molyneaux L, D'Souza M, Twigg SM, Wu T (2020). Young-onset type 2 diabetes and younger current age: increased susceptibility to retinopathy in contrast to other complications.. Diabet Med..

[27] Cho Y, Park HS, Huh BW, Seo SH, Seo DH, Ahn SH (2022). Prevalence and risk of diabetic complications in young-onset versus late-onset type 2 diabetes mellitus.. Diabetes Metab..

[28] Aulich J, Cho YH, Januszewski AS, Craig ME, Selvadurai H, Wiegand S (2019). Associations between circulating inflammatory markers, diabetes type and complications in youth.. Pediatr Diabetes..

[29] Wijayaratna S, Lee A, Park HY, Jo E, Wu F, Bagg W (2021). Socioeconomic status and risk factors for complications in young people with type 1 or type 2 diabetes: a cross-sectional study.. BMJ Open Diabetes Res Care..

[30] Haynes A, Sanderson E, Smith GJ, Curran JC, Maple-Brown L, Davis EA (2021). Demographic and clinical characteristics of a population-based pediatric cohort of type 1 and type 2 diabetes in Western Australia (1999-2019).. Pediatr Diabetes..

[31] Carino M, Elia Y, Sellers E, Curtis J, McGavock J, Scholey J (2021). Comparison of Clinical and Social Characteristics of Canadian Youth Living With Type 1 and Type 2 Diabetes.. Can J Diabetes..

[32] Varley BJ, Gow ML, Cho YH, Benitez-Aguirre P, Cusumano J, Pryke A (2022). Higher frequency of cardiovascular autonomic neuropathy in youth with type 2 compared to type 1 diabetes: Role of cardiometabolic risk factors.. Pediatr Diabetes..

[33] Jaiswal M, Divers J, Urbina EM, Dabelea D, Bell RA, Pettitt DJ (2018). Cardiovascular autonomic neuropathy in adolescents and young adults with type 1 and type 2 diabetes: The SEARCH for Diabetes in Youth Cohort Study.. Pediatr Diabetes..

[34] Yeow TP, Aun ES, Hor CP, Lim SL, Khaw CH, Aziz NA (2019). Challenges in the classification and management of Asian youth-onset diabetes mellitus- lessons learned from a single centre study.. PLoS One..

[35] Pleniceanu O, Twig G, Tzur D, Gruber N, Stern-Zimmer M, Afek A (2021). Kidney failure risk in type 1 vs. type 2 childhood-onset diabetes mellitus.. Pediatr Nephrol..

[36] Middleton TL, Chadban S, Molyneaux L, D'Souza M, Constantino MI, Yue DK (2021). Young adult onset type 2 diabetes versus type 1 diabetes: Progression to and survival on renal replacement therapy.. J Diabetes Complications..

[37] Ferm ML, DeSalvo DJ, Prichett LM, Sickler JK, Wolf RM, Channa R (2021). Clinical and Demographic Factors Associated With Diabetic Retinopathy Among Young Patients With Diabetes.. JAMA Netw Open..

[38] Ek AE, Samuelsson U, Janson A, Carlsson A, Elimam A, Marcus C (2020). Microalbuminuria and retinopathy in adolescents and young adults with type 1 and type 2 diabetes.. Pediatr Diabetes..

[39] Inge TH, Laffel LM, Jenkins TM, Marcus MD, Leibel NI, Brandt ML (2018). Comparison of Surgical and Medical Therapy for Type 2 Diabetes in Severely Obese Adolescents.. JAMA Pediatr..

[40] TODAY Study Group (2021). Postintervention Effects of Varying Treatment Arms on Glycemic Failure and beta-Cell Function in the TODAY Trial.. Diabetes Care..

[41] Laffel LMB, Tamborlane WV, Yver A, Simons G, Wu J, Nock V (2018). Pharmacokinetic and pharmacodynamic profile of the sodium-glucose co-transporter-2 inhibitor empagliflozin in young people with Type 2 diabetes: a randomized trial.. Diabet Med..

[42] Tamborlane WV, Laffel LM, Shehadeh N, Isganaitis E, Van Name M, Ratnayake J (2022). Efficacy and safety of dapagliflozin in children and young adults with type 2 diabetes: a prospective, multicentre, randomised, parallel group, phase 3 study.. Lancet Diabetes Endocrinol..

[43] Shankar RR, Zeitler P, Deeb A, Jalaludin MY, Garcia R, Newfield RS (2022). A randomized clinical trial of the efficacy and safety of sitagliptin as initial oral therapy in youth with type 2 diabetes.. Pediatr Diabetes..

[44] Tamborlane WV, Barrientos-Perez M, Fainberg U, Frimer-Larsen H, Hafez M, Hale PM (2019). Liraglutide in Children and Adolescents with Type 2 Diabetes.. N Engl J Med..

[45] Tamborlane WV, Bishai R, Geller D, Shehadeh N, Al-Abdulrazzaq D, Vazquez EM (2022). Once-Weekly Exenatide in Youth With Type 2 Diabetes.. Diabetes Care..

[46] Singhal S, Kumar S (2021). Current Perspectives on Management of Type 2 Diabetes in Youth.. Children (Basel)..

