# Supplementary material for: Complications and Treatment of Early-Onset Type 2 Diabetes
Source: Int J Endocrinol Metab. 2023 Aug 6;21(3):e135004. doi: 10.5812/ijem-135004 (PMC10676662; doi:10.5812/ijem-135004)
Supplement: ijem-21-3-135004-s001.pdf [file ijem-21-3-135004-s001.pdf]

Appendix:

Searched keywords in titles and abstracts included:

((Diabetes Type II) OR (Diabetes Type two) OR (Diabetes Type 2) OR (Diabetes Adult-Onset) OR (Diabetes Adult Onset) OR (Type II diabetes) OR (Type two diabetes) OR (Non-Insulin Dependent Diabetes) OR (Type 2 diabetes)) AND ((Young-onset) OR (Young onset) OR (Youth-onset) OR (Youth onset) OR (Young*) OR (Youth*) OR (Adult onset) OR (Adult-onset) OR (Adult*) OR (Young adult*) OR (Young adult-onset) OR (Young adult onset) OR (Adolescent*) OR (Adolescence*) OR (Adulthood) OR (Teenager*) OR (Pediatric*) OR (Children) OR (age <40)) AND (((Complication*) OR (Micro vascular) OR (Macro vascular) OR (Micro-vascular) OR (Macro-vascular) OR (Retinopathy) OR (Nephropathy) OR (Neuropathy) OR (Microvascular*) OR (Macrovascular*)) OR ((Therapeutic*) OR (Treatment*) OR (Drug*) OR (Medication*) OR (Management*)))
